# Extension for Community Healthcare Outcomes Based Telementoring of Physicians for Dementia–Effectiveness in India

**DOI:** 10.3389/fpsyt.2022.869685

**Published:** 2022-05-23

**Authors:** Shruti Nair, Preeti Sinha, Prabhat Chand, Prashant Sahu, Naga VSS Gorthi, Mathew Varghese, P. T. Sivakumar

**Affiliations:** Department of Psychiatry, National Institute of Mental Health and Neurosciences (NIMHANS), Bengaluru, India

**Keywords:** ECHO, telementoring, dementia, training, primary care

## Abstract

**Objective:**

The study aimed to evaluate the effectiveness of a program based on the telementoring model [Extension for Community Healthcare Outcomes (ECHO)] for primary care physicians in diagnosing and treating patients with dementia.

**Method:**

The ECHO model was adapted through 12 live sessions of 2 h every 2 weeks consisting of a didactic presentation by the expert, peer-led new case discussions, and follow-up discussions. In addition, there were 10 h of self-paced e-learning and interim assignments. The impact was examined by noting participation, session ratings, monthly clinical reports, and comparing knowledge and competency scores before and after the course.

**Results:**

Among the 63 participants, 39.7% attended at least 80% of the sessions; completing the program successfully. The ratings for all sessions ranged from “good” to “excellent.” The paired sample *t*-test revealed a statistically significant improvement (*p* < 0.001) in self-rated skills and confidence in diagnosing and treating dementia with an effect size of 1.25 and 1.37, respectively. No change in the knowledge score was observed throughout the course. A considerable increase in dementia-related clinical practice was observed during four monthly summary of clinical cases. Due to the limited data of monthly reports during the COVID pandemic, no statistical analysis was attempted.

**Conclusion:**

The ECHO model appears to have a positive immediate impact on the clinical ability of primary care physicians to diagnose and treat dementia. Its direct impact on patient health and at the community level should be aimed at in future studies.

## Introduction

Population aging is gradually taking the global form. In some low- and middle-income countries (LMIC), the proportion of older people could double in 50 years, which had happened over 150–200 years in high-income countries (HIC). In India, too, people over the age of 60 years are projected to increase from 8.6% in 2011 to 19.5% of the total population in 2050, probably leading to an absolute number of 320 million people. With the less availability of resources at the national level and the slower recognition and response of the government to the dependence of older people, this faster rate of growth of the aging population amplifies the scarcity of resources for elderly healthcare (1). A significant health problem in the elderly is dementia, recognized as a public health priority by the World Health Organization (WHO), calling for global action in 2015 (2). The current prevalence of dementia worldwide, 50 million, is expected to increase approximately three times to 131 million in 2050, with the highest prevalence in LMIC (3). In India, the estimated prevalence of 3.7 million in 2010 is projected to rise to 14.32 million by 2050 (4).

Research reveals a delay in the diagnosis of dementia, a delayed initiation of dementia-specific medications, and inadequate pharmacological and psychosocial management within the primary care system (5). The treatment gap in dementia care is much more significant in LMICs with a deficiency in the development of policies, care plans and their implementation at the community level (6). An efficient primary care system with coordination between primary and specialist care services is required to reduce the treatment gap (2). The Dementia India Report 2010 reveals less than 0.2 human resource personnel, including psychiatrists, neurologists, nurses and social workers per 100,000 population in India (4). One way of integrating dementia care into primary care is to develop locally relevant material for primary care physician training and improve referral patterns (7). This can be explored with telehealth, which has been used to train and support caregivers of dementia patients remotely over the last few years (8–10).

The Project Extension for Community Healthcare Outcomes (ECHO) is an innovative model based on the concept of telehealth to bridge the gap between a specialist and a primary care physician. Unlike telemedicine, where the specialist is in charge of patient care, the ECHO model trains and enables the primary care physician to provide specialist care in a community setting. It follows a virtual hub-and-spoke educational model using telementoring, case-based discussions, and didactics to access the best evidence-based practices and improve the expertise in specialty conditions of primary care physicians (11). A recent systematic review of 39 studies evaluating the impact of Project ECHO on 17 different health conditions revealed the cost-effectiveness of this model in reducing patient travel for specialist care and thus positively impacting clinical outcomes. Preliminary data show that it also facilitates a positive change in the knowledge, competence, and performance of primary care physicians to manage the respective disorder (12).

Project ECHO has specifically contributed to the health of older adults only through a few programs in the United States. ECHO Geriatrics, which trained two different groups of primary care physicians in general geriatric medicine from two other regions, reported a significant improvement in self-reported geriatric knowledge and competencies and practice behaviors centered on geriatrics (13, 14). ECHO AGE aimed to link experts in the management of behavioral disorders of dementia with nursing home care by video conferencing. The evaluation of the outcome revealed an improvement in clinical outcome (74% vs. 20%, *P* < 0.03) with a reduced hospitalization (15). The ECHO model has only recently been used to provide clinical skills in dementia through the program called “Dementia 360” aimed at rural clinicians. The program recorded an improvement in the level of comfort of physicians in diagnosing and treating people with dementia (16).

Thus, the ECHO model has the potential to reduce the treatment gap for the elderly population with dementia in a resource-poor country such as India, where there are very few geriatric specialists. This model has been implemented in India through ECHO-India for various health conditions. NIMHANS ECHO, one of the key ECHO hubs in India, pioneered the adaptation of the ECHO model for mental health and addiction care and showed significant success in reducing the urban-rural divide in treatment facilities in at least three different states (17–20). Therefore, the Dementia ECHO certificate program for primary care clinicians was conducted at the NIMHANS ECHO center. The study aimed to evaluate the effects of the telementoring model (ECHO) through this program on the satisfaction, knowledge and competence of primary care physicians in the diagnosis and treatment of patients with dementia. The study also examined the immediate effect of the model on the behaviors related to the clinical practice of the participating clinicians.

## Materials and Methods

### Design and Participants

The study was based on training data from a digital (online) certificate course on dementia at the NIMHANS Digital Academy conducted for 10 months between October 2019 and July 2020. Anonymity was maintained and informed consent was obtained from all participants. Institutional Ethics Committee approval was granted. All participants were physicians who completed at least MBBS and were certified in any program on the basics of mental health (e.g., Diploma in community mental health run by the NIMHANS Digital Academy) or who underwent a 2-/3-year residency course in psychiatry. All of them also needed to work with older adults at the primary care level, some of whom are people with memory problems or are being diagnosed with dementia. Doctors who were specialized in any specific field of medicine or surgery and worked with patients with complaints restricted to the respective field were excluded.

### Assessments

1. During the participant enrollment process, we collected details about their qualification, clinical work profile, and clinical setting.
2. Online (internet-based) assessment: It consisted of questions to assess the knowledge, clinical skills and confidence of participants in the screening, diagnosis and treatment of patients with dementia. This questionnaire was initially administered and then after completing the entire course of live sessions. All sections used the self-rated assessment pattern. For example, participants had to select one of the five options related to the frequency of the clinical skill suggested as “I *routinely ask about memory complaints to my elderly patients.*” The section “Knowledge” included a theoretically based evaluation such as “Which *of the following is the preferred measure to handle psychiatric/behavioral symptoms that develop in dementia*” and providing options related to specific antipsychotics and behavioral management for selection.
3. Monthly summary of clinical work related to older adults: This information was collected through an online form at the end of each month from October 2019 to July 2020. It examined the clinical aggregates of older adults seen by the doctors in their clinic and, among them, the number of older adults with memory problems, diagnosed with any dementia, MCI or Alzheimer’s disease, and the treatment provided for dementia in terms of cholinesterase inhibitors, psychotropics, and non-pharmacological treatment.
4. Session ratings: Participants were asked to anonymously rate each session using a five-point internet-based Likert scale at the end of the live session. The ratings considered were as follows: “poor, average, good, very good, and excellent.”
5. Attendance was recorded for all live sessions.

### Procedure

Participants underwent a digital training titled “Dementia: Diagnosis and treatment for physicians” conducted by a team of psychiatrists providing clinical services and training for geriatric psychiatry at NIMHANS, an academic institute with tertiary-level healthcare facilities. Following the hub and spoke model, our team had 4 key experts on dementia, PSi, MV, PTS, and SG. They work primarily with geriatric patients with dementia and other psychiatric disorders. We also had PC, SN, and PSa with expertise in implementation of an ECHO based program. The dementia expert group finalized the course curriculum after peer review by dementia experts outside of NIMHANS. It covered the screening for cognitive problems, the history and mental status examination of an older adult, the structured assessment of dementia, the salient characteristics of mild cognitive impairment and Alzheimer’s disease, distinguishing them from other dementias, the management of behavioral and psychological symptoms in dementia, the utility of cognitive enhancers and non-pharmacological treatment in dementia, and prevention of dementia.

This certificate course was conducted in association with Project ECHO–India. Participants were briefed on the principles and process of ECHO, the program curriculum, and the requirements for course certification before starting it. They were encouraged to ask questions, although no workshops were conducted to train on these aspects in advance. The curriculum was covered through a blended teaching technique that included virtual live video conference sessions every 2 weeks and self-paced e-learning. Live sessions were held for 2 h on the first and third Mondays and accounted for 24 h with 12 sessions. In self-paced e-learning, participants were involved through Google Classroom for a total of 10 h. Curriculum-based resource materials, assignments, and self-assessments were administered through this platform. This asynchronous e-learning is an enhancement to the synchronous learning of ECHO tele-clinic. It has been adapted to all programs of NIMHANS ECHO with the objective of having more self-controlled learning, specifically factual once (20, 21).

The live session had a didactic presentation on a predetermined topic related to one of the domains mentioned above. It lasted 30–45 min, including the topic-related interaction between the expert and the participants. The remaining time of the 2 h live session was dedicated to case discussion. Here, one or two participants presented their respective deidentified case of suspected or diagnosed dementia, which they noted in clinical work, and faced challenges in diagnosis and treatment. The specific case presentation form was developed to cover all relevant history and examination of cognitive, psychiatric, and medical aspects together with a structured cognitive evaluation. Then a discussion was followed with the expert and other participants and ended with the plan for further evaluation and management of the case. The cases discussed were also followed up in subsequent sessions to see the result of the planned recommendations. All sessions were conducted using a web-based videoconferencing application that is compliant with the privacy and protection of health information according to the “Digital Information Security in Healthcare Act (DISHA), 2018” of the Government of India (22). All live sessions were also attended by one of the members of the Project ECHO India team, exclusive of the NIMHANS ECHO team.

The outcome evaluation of the course was performed with the Moore’s continued medical education (CME) evaluation framework (23).

– Level 1, Participation: A minimum of 80% attendance for live sessions was taken as one of the criteria for awarding certificates. The proportion of participants who achieved this criterion was noted.
– Level 2, Satisfaction: The satisfaction of the participants was assessed by an anonymous rating of each live session provided by them.
– Level 3, Learning and Knowledge: To receive certification, the participant had to secure a minimum of 80% in all interim assignments in 5 attempts over 2 weeks and present 2 cases during live sessions or otherwise to experts. Additionally, knowledge was assessed using the online questionnaire conducted before and after the course.
– Level 4, Competence: Participants conducted a self-assessment of clinical skills and confidence using the online assessment questionnaire before and after the course.
– Level 5, Performance: This was assessed using a monthly summary of the clinical cases seen by the participants. Details are described above.

### Statistical Analysis

Normality tests were applied to examine the distribution of the data. The frequency distribution of the demographic characteristics of the attended and accredited groups was compared with the chi-square to find any significant differences between the two groups. The knowledge, skills, confidence, and interest scores in learning were summarized in mean and standard deviation. The Paired Samples *t*-test was used to compare scores before and after the course. Data were evaluated at a 0.05 significance level.

## Results

### Description of the Sample

A total of 108 physicians had expressed interest in attending the program. Among them, 72 were eligible for participation in the program and 63 attended one or more sessions. 39.7% (*n* = 25) of these 63 physicians completed the program and were accredited. 53 (84.1%) physicians had previously attended one or more ECHO programs (related to mental health aspects). The sociodemographic characteristics of both groups are described in Table 1. An apparent difference was observed in the age distribution, academic qualifications and job profile. A quarter of the participants who attended were in the age group of 21–30 years. But among the accredited participants, all were over 30 years old. Although 9 (14.3%) of the participants were in residency, only one of them completed the program successfully.

**TABLE 1:** Demographic characteristics of certified participants.

Demographic characteristics		Accredited participants (Total = 25); *N* (%)	Attended participants (Total = 63); *N* (%)
Age	21–30	0(0%)	15(23.8%)
	31–40	11(44%)	24(38.1%)
	41–50	6(24%)	11(7.5%)
	51 and above	8(32%)	13(20.6%)
Sex	Male	19(76%)	51(81%)
	Female	6(24%)	12(19%)
Employment	Government	19(76%)	48(76.2%)
	Private	6(24%)	15(23.8%)
Work setting	Rural	4(16%)	16(25.4%)
	Semi-urban	7(28%)	12(19%)
	Urban	14(56%)	35(55.6%)
Work profile	Consultant Physician	3(12%)	10(15.9%)
	Consultant Psychiatrist	5(20%)	11(17.5%)
	Medical Officer (PHC/District hospital)	16(64%)	33(52.4%)
	Resident	1(4%)	9(14.3%)
Qualification	MBBS	6(24%)	28(44.4%)
	Post-graduate	19(76%)	35(55.6%)

Similarly, MBBS graduates had a higher proportion in the enrolled group (44.4%) compared to the accredited group (24%). However, the chi-square analysis did not reveal significant differences between the two groups for any characteristics. Most of the attending physicians and those certified were men and worked in government hospitals in an urban setting. The largest work profile in both groups was the medical officers of primary health centers or district hospitals.

Participants were asked about the expectations of the program prior to its onset. The main expectations were as follows.

– Improve skills in screening for dementia.
– Learn how to diagnose and treat patients with dementia.
– To understand cognitive problems in older adults.
– To learn specifically about dementia due to Alzheimer’s disease.

### Evaluation of the Course Outcome

**FIGURE 1:**
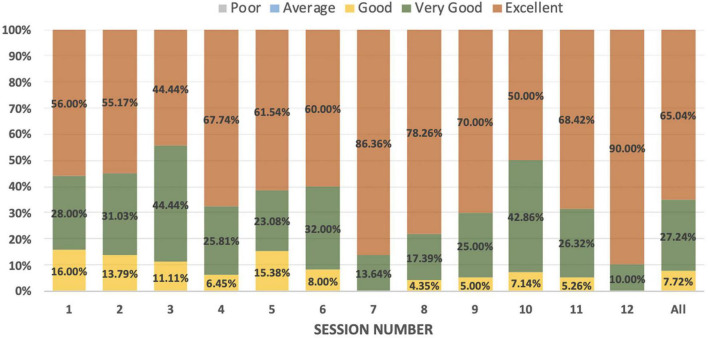
Distribution of rating for online sessions.

a. Participation: 52.4% (*n* = 33) of the physicians attended at least 6 of the 12 sessions, that is, 50 and 39.7% (*n* = 25) attended 10 sessions, that is, 80%.
b. Satisfaction: 45–90% of participants in different sessions gave an “excellent” rating. Other scores received for the sessions were “very good” and “good.” None of the sessions received a “poor” rating from any participant. The detailed distribution is presented in Figure 1. The sessions with the “excellent” rating from about 70% or more of the participants were related to the behavioral symptoms associated with dementia and the pharmacological and non-pharmacological treatment of dementia. The last session focused on giving a glimpse of all previous sessions through case vignettes and received the “excellent” rating from 90% of the participants.

c. Learning and Competence: The case presentation and interim assignments were satisfactorily completed for certification by 25 participants. The cases presented ranged from mild cognitive impairment (MCI) to severe dementia. Many of the cases were presented to discuss the management of associated behavioral and psychological symptoms. There were also difficulties in diagnosing MCI, differentiating dementia from psychiatric disorders, and addressing metabolic factors associated with dementia. Otherwise, the type of dementia was limited to Alzheimer’s disease and Vascular dementia. The cases presented also had comorbidities ranging from diabetes, hypertension, and nutritional deficiency to ischemic heart disease, stroke, and psychiatric disorders such as depressive disorder, bipolar affective disorder, and schizophrenia. These comorbidities presented challenges in pharmacological and non-pharmacological treatments and were addressed accordingly.

**FIGURE 2:**
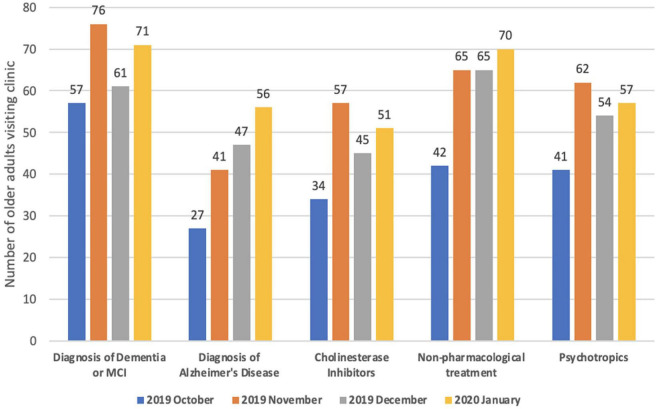
Clinical profile of patients with dementia visiting physician’s clinic–Monthly aggregate of all participants (physicians).

**FIGURE 3:**
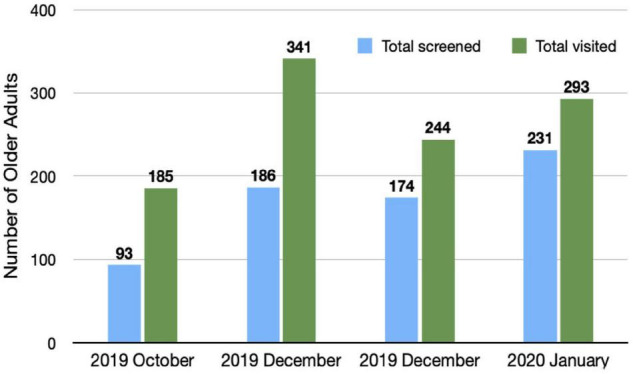
Older adults visiting physician’s clinic and screened for memory problems: monthly aggregate of all participants (physicians).

All accredited participants completed the pre-and post-assessment questionnaires. The paired sample *t*-test revealed a statistically significant improvement in the skills and confidence of certified participants with an effect size of 1.25 and 1.37, respectively. The level of knowledge and interest in learning did not change from the pre- to post-phase of the program.

d. Performance (Figures 2, 3): We present the monthly summary of the clinical profile of the cases seen by the participants from October 2019 to January 2020 only. For months after then, the number of consultations was very restricted due to lockdown and other restrictions of movement posed during the COVID-19 pandemic. We focused only on patients with dementia. In the first 4 months of the monthly report, a substantial increase in patients diagnosed with dementia (from 57 to 71) and dementia due to Alzheimer’s disease alone (from 27 to 56) was observed. Similarly, a higher proportion of older adults who visited the clinic of all participants were asked about memory problems in January 2020 (*n*/*N* = 231/293) compared to those in October 2019 (*n*/*N* = 93/185). There was also a gradual increase in patients managed with pharmacological and psychosocial treatments during these 4 months.

## Discussion

With the advancement in videoconferencing technology and its ease of availability in recent years, the scope of education and training through telementoring has gained momentum in the medical and surgical fields (24, 25). We conducted a digital certificate course program based on a telementoring model (ECHO) to train primary care physicians on the diagnosis and treatment of dementia. The program was conducted through the NIHMANS Digital Academy between October 2019 and July 2020. It can be considered the first effort to bring the benefits of the ECHO model to dementia care in a developing country. The current study evaluated the effect of this program on the knowledge, confidence, and clinical skills of primary care physicians in dementia and on the clinical profile of elderly patients seen by them. The objectives of the program matched the expectations of the participants before joining the course.

The results showed a significant improvement, that is, approximately 1.5 times, in the clinical skills and confidence of accredited physicians in the screening, diagnosis, and treatment of dementia after completion of the program. The results reflect the comprehensiveness of the course in providing clinically relevant material. The regular provision of assignments would have helped participants develop self-reliance to handle elderly with cognitive problems, which is also reflected in the monthly reports of the participants showing a trend of improvement in clinical practice. For 4 months, there was an increase in the number of older adults screened for memory disturbances identified with Alzheimer’s disease and other dementias and the use of pharmacological and psychosocial methods in treatment. Other studies based on the ECHO model dealing with complete geriatric medicine (13, 14) or specific areas such as dementia (16) and geriatric mental health (26) have also noted an increase in confidence, self-assessed competence, clinical skills, and practice. However, none of them objectively evaluated the clinical practice of physicians during the program. These programs also did not provide interim assignments and, except for one program (13), probably did not supplement live sessions with self-paced e-learning resources.

We diligently followed the ECHO model of telementoring (11). A total of 84% of the participants had previous experience with the ECHO model. Additionally, the team provided information related to the concept, process, and steps of ECHO before the sessions started. The program included 2-h video conference sessions and self-paced e-learning with study material; the former had a didactic presentation by the expert and a virtual live case discussion. In addition, the participants had the opportunity to discuss the follow-up of their presented cases after implementing the expert’s suggestions. Participants also performed a clinical evaluation and cognitive screening of elderly patients visiting their clinics with the *pro forma* provided in this training program. Instead of a weekly schedule as in the original ECHO model, this program had fortnightly sessions to provide adequate time to grasp self-learning materials and assignments. This frequency has been adopted in some ECHO programs, including dementia care-related programs (27). Overall, our fidelity to the original ECHO model can be considered for 3 of 4 criteria that amounts to mid-level fidelity (12). And we made sure that all aspects of fidelity were directly assessed by the Project ECHO India team.

There was no significant increase in the knowledge score after completing the course. The mean scores for the knowledge before and after the course were above 50%. The program probably helped physicians improve the clinical application of already acquired knowledge, and 12 sessions may not have been enough to improve theoretical knowledge. Interest in learning was relatively high before the start of the program, with a mean score of 30.7 out of a total score of 35. Interest in attending the program was maintained at a similar level, which validates the good quality of the program. This observation is further supported by the higher scores (4 or 5) received in 80–90% of the responses in all sessions. The participants liked the sessions related to the treatment aspects and the didactics presented through case vignettes.

According to Moore’s CME evaluation framework, this program achieved good results in satisfaction, learning, competence, and the participants’ performance (23). The 25 accredited participants (39.7%) had attended at least 10 (83.3%) of the 12 sessions. The number of participants who attended at least 50% of the sessions was moderate, 33 (52.4%). The average participation has varied in different ECHO programs. A couple of ECHO programs on geriatric care had 69–79% of the participants attending more than half of the total sessions (14, 16). On the contrary, some have reported a lower average participation of 2.7 out of 15 sessions. One of the possible reasons for the reduced participation in this program could be the onset of the COVID-19 pandemic in the latter half. Many physicians were asked to join COVID-19 related duties and would have found it difficult to attend the program. COVID-19 related duties also affected their visits to regular clinics, as noted in the monthly reports. It is also possible that few participants may not have realized the program requirements at the beginning of the course and eventually could not complete the requirements, especially residents, as only 1 out of 9 participating residents got certification.

The program was designed to enable physicians to diagnose and provide comprehensive treatment for patients with dementia. This approach is similar to the earlier Dementia 360 telementoring program conducted for clinicians in the Oregon state of the United States (16). Other programs intended for general geriatric medical care or geriatric mental health included other medical professionals, such as nurses, physician assistants, and pharmacists. As in this program, all of these programs have allowed residents and physicians of different specialties (13, 14, 16, 26).

## Limitations and Future Directions

This study is based on only one cycle of the program. Therefore, the program had a restricted number of accredited participants, i.e., 25. A smaller proportion (40%) of the total participants were accredited, which led to a small sample size. Increasing the number of accredited participants at the cost of a possible effective course and fidelity to the ECHO program was not preferred. This small sample prevented us from effectively analyzing the impact of the program on various parameters and could not have enough power to detect the change in other aspects of the evaluation of the program. Changes related to COVID-19 further limited long-term follow-up of physician clinical activities. Immediate changes in this program related to patient health and community level, levels 6 and 7 of Moore’s expanded CME framework, were not considered. This change would require regular updates on the specific patient outcomes of accredited participants. In this regard, adding an e-consult, such as constant support and feedback to the accredited group from experts through online consultation, may help achieve the outcome. As dementia involves subjective evaluation of behavioral problems, an e-consulting facility for physicians to reach out could help them improve their clinical skills and help monitor patient health more effectively. Additionally, community-level changes would require an increase in the number of accredited participants from the confined region, which can be planned in later cycles of the dementia ECHO program. Including the training of other mental health service providers, such as home care providers and psychiatric social workers, in our program would improve community-level outcomes. This study could also encourage the use of ECHO models to train accredited physicians in this program on other dementias and various other aspects of geriatric health.

## Conclusion

The ECHO model appears to be a promising innovation to bridge the treatment gap in dementia. In a country like India, where the prevalence of dementia is expected to increase drastically, but experts are limited, the ECHO model shows that videoconferencing-based telelearning and clinics can train and empower a large number of primary care physicians remotely in different parts of the country. The collaborative network based on the ECHO model of experts and primary care physicians should be built and further evaluated for its impact at the patient and community level.

## Data Availability Statement

The raw data supporting the conclusions of this article will be made available by the authors, without undue reservation.

## Ethics Statement

The studies involving human participants were reviewed and approved by Institute Ethics Committee, Behavioural Sciences, National Institute of Mental Health and Neurosciences (NIMHANS). The patients/participants provided their written informed consent to participate in this study.

## Author Contributions

PSi oversaw the training program mentioned here, supported by the equal contribution of SN and PSa, MV, PTS, PC, and NG. PSi and SN prepared the manuscript, with support from the other authors. All authors contributed to the article and approved the submitted version.

## Conflict of Interest

The authors declare that the research was conducted in the absence of any commercial or financial relationships that could be construed as a potential conflict of interest.

## Publisher’s Note

All claims expressed in this article are solely those of the authors and do not necessarily represent those of their affiliated organizations, or those of the publisher, the editors and the reviewers. Any product that may be evaluated in this article, or claim that may be made by its manufacturer, is not guaranteed or endorsed by the publisher.
